# Austrian Syndrome – A Devastating Osler’s Triad: Case Report and Literature Review

**DOI:** 10.7759/cureus.4486

**Published:** 2019-04-17

**Authors:** Rastko Rakočević, Sara Shapouran, Kathleen M Pergament

**Affiliations:** 1 Internal Medicine, University Hospital - Rutgers New Jersey Medical School, Newark, USA; 2 Neurology, University Hospital - Rutgers New Jersey Medical School, Newark, USA

**Keywords:** austrian syndrome, acute bacterial meningitis, embolic stroke, streptococcal infection, infectious endocarditis, streptococcus pneumoniae, septic arthritis, osler's triade

## Abstract

Austrian syndrome is a rare triad of meningitis, pneumonia, and endocarditis caused by Streptococcus pneumoniae. We present a case of the Austrian syndrome in the oldest patient in the reviewed literature, with no other classically described risk factors. She had an unusual initial presentation and microorganism portal of entry. Her hospital course was complicated by the diagnosis of monoclonal gammopathy, septic knee joint, septic brain emboli and respiratory failure. We also provide an extensive review of available literature of this commonly unrecognized entity.

## Introduction

The rare triad of meningitis, pneumonia, and endocarditis caused by Streptococcus pneumoniae (S. pneumoniae) is known as Austrian syndrome (Osler’s triad) and is associated with high morbidity and mortality rates despite aggressive therapeutic management.

## Case presentation

A 76-year-old active and independent woman with a history of diabetes mellitus, hypertension and chronic cervical and lumbar degenerative disease presented to our hospital with a three-day history of headache and minimally altered mental state (AMS, rude and aggressive per family). She was afebrile on presentation and initial laboratory and radiological workup was negative for urinary tract infection (UTI) or pneumonia, but did reveal white blood cell count (WBC) of 11.100/µl. She was observed without antibiotic therapy for two days. In the morning of the day three of admission she had WBC of 7.200/µl with 15% bands and was still afebrile. On physical exam three hours afterwards, she was completely disoriented with Glasgow Coma Scale score of nine (E2M5V2) and significant photophobia, was febrile to 103.2F but otherwise hemodynamically stable. Kernig’s and Brudzinski’s signs were equivocal. Computed tomography (CT) and magnetic resonance imaging (MRI) head, urinalysis, and chest X-ray showed no acute pathology. Image-guided lumbar puncture was unsuccessful due to diffuse structural spine changes. Intravenous ceftriaxone, vancomycin, ampicillin, acyclovir and dexamethasone were started for empiric meningitis treatment. Urine antigen and blood cultures results were positive for Streptococcus pneumoniae and antibiotic therapy was narrowed down to ceftriaxone on day three. A repeat chest X-ray revealed a new consolidation visualized at the right lung base, consistent with pneumonia. Even though fever resolved and leukocytosis improved, due to minimal mental status improvement, and based on the S. pneumoniae sensitivity from blood cultures, rifampicin was added to therapy from days 10 to 20 which correlated with limited but gradual clinical improvement. On day 17, follow-up MRI brain showed multiple new foci of restricted diffusion in the frontal and parietal lobes, consistent with septic emboli. Transthoracic echocardiogram revealed a new mobile mass on the aortic valve consistent with infectious endocarditis, confirming the diagnosis of Austrian syndrome. During hospitalization, the patient also developed septic knee arthritis, C1 spine subluxation, monoclonal gammopathy, and acute hypoxic respiratory failure requiring a short period of intubation. Ceftriaxone therapy was continued, and the patient’s mental status improved. She was discharged on day 31 and completed a total of six weeks of ceftriaxone treatment. One month later, she was admitted for AMS and UTI, treated with antibiotics with mental status improvement. One month after that, she returned with AMS, muscle twitching, and UTI, and was found to have a right frontal focal status epilepticus. MRI and CT head ruled out new infectious or inflammatory lesions. The patient was discharged to home hospice where she eventually died.

## Discussion

Austrian syndrome was first described in 19th century as the triad (Osler’s triad) of pneumonia, meningitis and endocarditis following pneumococcal infection, with a 75% mortality rate. This syndrome is more common in the setting of alcohol use disorder (historically, alcoholism completed the tetrad of associated conditions) and in men. Before the 1940s, when the introduction of the penicillin changed the epidemiology of susceptible infections, pneumococcus was responsible of 15-20% of all endocarditis cases. Currently, S. pneumoniae is the cause of fewer than 3% of endocardial infection [1,2] especially community-acquired cases [3]. S pneumoniae endocarditis confers a high risk of mortality without surgical treatment (greater than 60%) [3], with native aortic valve being the most frequent site of vegetation [3,4]. Vegetations are usually extensive and lead to frequent septic embolization [5] as in this case. Fewer than 1% of patients with pneumococcal endocarditis present with the classic Austrian triad [4]. Similarly, even though S. pneumoniae remains the most prevalent bacteria causing community acquired meningitis in adults, with high mortality (25%) and morbidity rates despite adequate antibiotic or corticosteroid treatment [6,7], endocarditis is rare. In a retrospective study with 80 cases of pneumococcal meningitis in the intensive care unit (ICU), only six patients developed endocarditis [6].

The most common microbial portal of entry leading to development of Austrian syndrome is proposed to be lungs, followed by endocarditis and meningitis [8,9], respectively. Regardless of the route of entry, the most common predisposing factors are alcohol use, male sex, and advanced age [4-11]. Moreover, the majority of patients have other systemic illnesses [10]. On presentation, our patient's only major risk factor for Austrian syndrome was advanced age; in fact, to our knowledge, she is the oldest patient with Austrian syndrome on literature review. Although the condition was diagnosed during this hospitalization, our patient’s monoclonal gammopathy likely put her at increased risk for developing the triad. However, since the patient’s family declined bone marrow biopsy, the true diagnosis remains a mystery. We found a single case report of patient being diagnosed with multiple myeloma during the hospitalization for Austrian syndrome, who died soon after the diagnosis [11]. It is proposed that gammopathies cause impairments in complement activation, leading to an immunocompromised state [12]. Interestingly, our patient was vaccinated with pneumococcal polysaccharide vaccine (PPSV-23) but had never received pneumococcal conjugate vaccine (PCV-13). In addition to the incomplete immune protection conferred by PPSV-23, it is possible that monoclonal gammopathy, diabetes, or other undiagnosed systemic disease led to an impaired immunity response to vaccine, which resulted in insufficient protection [13].

Even though penicillin has been the preferred therapy for S. pneumoniae meningitis and endocarditis for decades, many strains of S. pneumoniae have developed significant penicillin resistance, resulting in the current standard of using third-generation cephalosporins (ceftriaxone or cefotaxime) in combination with vancomycin as initial first-line therapy for pneumococcal infection. Expert opinions also suggest treatment with rifampicin if the bacterial isolate shows resistance to the aforementioned cephalosporins [14,15]. Although there are mixed opinions about the role of corticosteroids in the treatment of meningitis, it has been shown [7,16] that early treatment with dexamethasone improves the clinical outcome in adults with pneumococcal meningitis, likely via reduction of the inflammatory response and restoration of hemodynamic stability in cases of disseminated pneumococcal infection, particularly in cases of septic shock. In pneumococcal endocarditis, valve replacement should be performed as soon as possible to avoid the development of cardiogenic shock [17] as only 17% of cardiac vegetations disappeared after appropriate antibiotic therapy [17,18].

## Conclusions

Our case highlights several valuable teaching points. Firstly, the diagnosis of meningitis must be considered even with minimal alteration of mental status with or without leukocytosis, especially if bandemia is present. This was initially unrecognized in our patient, leading to a delay in the diagnosis and treatment of meningitis. Secondly, since our patient exhibited mostly neurological symptoms without any concomitant major cardiopulmonary symptoms, at least during the first week of her hospitalization, and did not have most of the classic risk factors, Austrian syndrome was not considered as a diagnosis until endocarditis and pneumonia became apparent; fortunately, this delay did not change clinical management of the patient, as her family declined aggressive management such as aortic valve replacement. Of note, to our knowledge, our patient is the oldest case of Austrian syndrome in the current literature. Even though our patient was on appropriate antimicrobial therapy based on bacterial blood culture susceptibility, her clinical mental status only significantly improved when rifampicin was added to the regimen. Through this case, we wish to emphasize the importance of having a high degree of clinical suspicion for Austrian syndrome in patients with S. pneumoniae infection, and certainly a higher degree of clinical suspicion in those with any of the known predisposing risk factors.
